# Mapping the evidence on factors related to postpartum contraception among sub-Saharan African immigrant and refugee women in the United States of America: A scoping review protocol

**DOI:** 10.1371/journal.pone.0304222

**Published:** 2024-05-29

**Authors:** Comfort Z. Olorunsaiye, Mariam A. Badru, Augustus Osborne, Hannah M. Degge, Sanni Yaya

**Affiliations:** 1 Department of Public Health, Arcadia University, Glenside, PA, United States of America; 2 College of Public Health and Health Professions, University of Florida, Gainesville, FL, United States of America; 3 Department of Biological Sciences, School of Environmental Sciences, Njala University, PMB, Freetown, Sierra Leone; 4 Department of Health and Education, Coventry University, Scarborough, United Kingdom; 5 School of International Development and Global Studies, University of Ottawa, Ottawa, Canada; 6 The George Institute for Global Health, Imperial College London, London, United Kingdom; Caleb University, NIGERIA

## Abstract

**Background:**

Postpartum contraception is essential to sexual and reproductive health (SRH) care because it encourages healthy spacing between births, helps women avoid unwanted pregnancies, and lessens the risks of health problems for mothers and babies. Sub-Saharan African immigrant and refugee populations are rapidly increasing in the United States, and they come from a wide range of cultural, linguistic, religious, and social origins, which may pose challenges in timely access to culturally acceptable SRH care, for preventing mistimed or unwanted childbearing. The objective of this scoping review is to assess the extent of the available literature on postpartum contraception among sub-Saharan African immigrant and refugee women living in the United States.

**Methods:**

We developed preliminary search terms with the help of an expert librarian, consisting of keywords including birth intervals, birth spacing, contraception, postpartum contraception or family planning, and USA or America, and sub-Saharan African immigrants, or emigrants. The study will include the following electronic databases: PubMed/MEDLINE, PsycINFO, CINAHL, EMBASE, and the Global Health Database. The sources will include studies on postpartum care and contraceptive access and utilization among sub-Saharan African immigrants living in the US. Citations, abstracts, and full texts will be independently screened by two reviewers. We will use narrative synthesis to analyze the data using quantitative and qualitative methods. Factors associated with postpartum contraception will be organized using the domains and constructs of the PEN-3 Model as a guiding framework.

**Conclusion:**

This scoping review will map the research on postpartum contraception among sub-Saharan African immigrant and refugee women living in the US. We expect to identify knowledge gaps, and barriers and facilitators of postpartum contraception in this population. Based on the findings of the review, recommendations will be made for advocacy and program and policy development toward optimizing interpregnancy intervals in sub-Saharan African immigrants living in the US.

**Trial registration:**

**Review registration** Open Science Framework: https://osf.io/s385j.

## Background

Postpartum contraception is essential to sexual and reproductive health (SRH) care because it helps women avoid unwanted pregnancies, reduces the risks of health problems for mothers and babies, and encourages healthy spacing between births [1]. The World Health Organization’s (WHO) Postpartum Family Planning Compendium (2013) provides guidelines for postpartum contraception, with recommendations for health care providers to initiate most postpartum contraception for most women from as early as within 48 hours of childbirth to six weeks—with a few other considerations for initiating certain methods after six weeks through the first 12 months postpartum (e.g., diaphragm for all women, and progestogen-only injections in breastfeeding women)—to prevent closely spaced and unintended pregnancies [2]. Similarly, within the first six weeks after childbirth, the American College of Obstetricians and Gynecologists (ACOG) recommends that women have access to various contraception options to prevent short-interval and/or unintended pregnancy [3]. Short interpregnancy intervals (IPIs), defined as an interval of less than 18 months between pregnancies [4], is a common occurrence among American women, yet many do not use effective postpartum contraception between pregnancies. Preterm delivery, low birth weight, and early gestational age are among pregnancy complications that are more likely to occur in women with shorter IPIs [5,6]. Healthy People 2020 aimed to decrease the number of infant mortality rates (IMRs) by 40 percent due to postpartum contraception usage [4]. Further, Healthy People 2030 aims to reduce the prevalence of pregnancies conceived within 18 months of a previous live birth, i.e., births with short IPI [7].

African immigrant and refugee populations, specifically populations from sub-Saharan Africa, are rapidly increasing in the United States, and they come from a wide range of cultural, linguistic, religious, and social origins [8]. Immigrant and refugee populations may have trouble using postpartum contraception and other preventative SRH care because of their prior experiences with SRH in their home countries, and relocation, migration, and resettlement experiences [9]. Evidence from other high-income migrant-receiving nations in the European Union and Asia [10], and a recent systematic review of five countries [11] have revealed many factors that contribute to low rates of maternal health care use among immigrant women. Language difficulties, immigrants’ exposure to prejudice and racism from healthcare professionals, women’s familiarity with maternal health care information, and the strain of cultural adjustment are all factors that impede the use of SRH services. Inadequate or no prenatal care, increased rates of stillbirths and infant death, and financial barriers [12] are some of the maternal health challenges the United Nations (UN) research finds common among immigrants across the European Union and Asia. British studies on immigrant birth outcomes show that infants delivered to Asian mothers had lower birth weights, and those born to Caribbean and Pakistani women have higher perinatal and postnatal death rates than the general population [13]. Other high-income countries, such as Norway, Japan, and Italy, have found similar results [11]. In the United States, the few studies available have found shorter IPIs in sub-Saharan African-born women compared to US-born women [14–16].

Low-income immigrant women in the US are particularly vulnerable because they typically lack the resources to pay for prenatal and postnatal care [17]. Medicaid, the biggest single-payer for obstetric care in the United States [18], is a lifeline for these women since it provides coverage for their pregnancies and limited postpartum care. However, many immigrant women are not covered by Medicaid, even though prenatal care is a prioritized preventive health treatment under the program [19]. Full (conventional) Medicaid benefits are not available to undocumented immigrants or documented immigrants in the country for less than five years [20]. Emergency Medicaid coverage, available in some states, which women must qualify for, limits coverage to just the direst circumstances, including hospitalization for delivery.

Understanding the factors influencing postpartum contraceptive use among sub-Saharan African immigrant and refugee women is vital for developing targeted interventions that address their unique needs. Sub-Saharan African immigrant and refugee women may face various challenges; culture and personal beliefs largely influence decisions regarding postpartum contraception. Some cultural norms and religious beliefs discourage modern contraceptive methods, endorsing more traditional methods or no contraception at all [21]. Access to healthcare services is a significant structural factor affecting postpartum contraception use among these women. Moreover, many sub-Saharan African immigrant and refugee women in the US face structural barriers to healthcare access, such as lack of insurance, language barriers, or unfamiliarity with the US healthcare system [22]. These barriers can limit these women’s ability to receive timely contraceptive counseling and access contraceptive services.

Financial considerations further impact the use of postpartum contraception. The cost of contraceptive methods, especially long-term methods like intrauterine devices (IUDs) and implants, may be prohibitive for some women, especially those that have low incomes and/or lack insurance coverage [23]. Although safety net family planning services via Title X funding may be available for uninsured or under-insured people, these services are commonly strained and many immigrants do not use them, sometimes due to lack of awareness or fear of jeopardizing their immigration status [24]. Educational level is also associated with postpartum contraception and immigrant and refugee women, with higher levels of education are more likely to use contraceptives than those with lower levels of education [25]. Education can improve women’s knowledge about contraceptive methods, their effectiveness, and where to access them. The role of healthcare providers is paramount in influencing postpartum contraceptive use. Effective, culturally sensitive contraceptive counseling by healthcare providers can encourage contraceptive use among immigrant women. However, health worker bias and lack of culturally sensitive health care services can hinder these efforts [26].

## Rationale for a scoping review

Sub-Saharan African immigrants account for the fastest growing group of Black immigrants in the US [27]. Moreover, according to recent data, sub-Saharan African immigrant women accounted for 5% of all live births in the US, and also have the highest birth rate among all immigrant groups in the US [28]. To our knowledge, there is no comprehensive review of the evidence on factors related to postpartum contraception among sub-Saharan African immigrant and refugee women in the US. Identifying the factors that contribute to lower rates of postpartum contraceptive use and higher rates of unintended and short interval pregnancies, which can have adverse health and socioeconomic consequences, is warranted. Therefore, the objective of this scoping review is to assess the extent of the available literature on postpartum contraception among sub-Saharan African immigrant and refugee women living in the United States. A scoping review is a suitable method to map the existing literature on this topic, identify knowledge gaps, and inform future research directions [29]. This review aims to build on existing research by assessing the evidence on factors related to postpartum contraception among sub-Saharan African immigrant and refugee women residing in the US. The review will identify barriers and facilitators to postpartum family planning services among immigrant and refugee people. By identifying the key factors influencing postpartum contraceptive use in this population, this study, when completed, can inform the development of interventions and policies promoting reproductive health equity for immigrant and refugee women living in the US.

## Methods

The protocol for this study was systematically developed using the Joanna Briggs Institute (JBI) guidelines for conducting scoping reviews to ensure replicability [30], and has been submitted to Open Science Framework (OSF) for registration. The review, when completed, will be reported according to the guidelines of the Preferred Reporting Items for Systematic Reviews and Meta-Analyses–Extension for Scoping Review (PRISMA-ScR) (S1 File) [31].

### Protocol design

The scoping review protocol was developed using the 2021 updated methodological guidelines of the Joanna Briggs Institute (JBI) [30]. Specifically, we followed the JBI multi-step process for protocol development, as described in the following paragraphs below.

### Title and review questions

This scoping review seeks to synthesize and summarize the available research on postpartum family planning among sub-Saharan African immigrant and refugee populations living in the United States, and to identify knowledge gaps in this area of research. The title and review questions were clarified and aligned with the purpose of the scoping review. Specifically, we ensured that key elements of the inclusion criteria, i.e., the population, concept, and context (PCC) are reflected in the title to help readers more easily identify the study [30,31]. The primary question that will guide the review is: What is the evidence on postpartum contraception among sub-Saharan African immigrant and refugee women living in the United States? In addition, three sub questions that will enable the research team to further explore specific attributes of the review’s population, concept, and context will be explored, as outlined below:

- What sub-Saharan African immigrant populations have been studied in the literature on postpartum contraception among sub-Saharan African immigrant and refugee women living in the US?
- What are the barriers to and facilitators of postpartum contraception from the perspectives of sub-Saharan African immigrant and refugee women and health care providers in the US?
- What is the evidence linking postpartum contraceptive counseling, access, and use to the prevention of short-interval births or unintended pregnancy among sub-Saharan African immigrant and refugee populations in the US?

### Inclusion criteria

As noted earlier, the eligibility criteria for this scoping review consist of three key elements: participants, concept, and context (PCC) [30].

#### Participants

The study particiants includes sub-Saharan African immigrant and refugee women of reproductive age (15–49 years) living in the US, and with at least one live birth. This age range aligns with the range used in population-based SRH surveys and databases [32]. Specifically, eligible studies will include participants that identify as immigrant or refugee individuals, born in a country in sub-Saharan Africa, and living in the US at the time of the study. Further, to better identify gaps in the literature, this protocol will also include sources that contain data on health care providers such as nurses, physicians, midwives, doulas, social workers, community health workers, and refugee resettlement staff, to provide relevant perspectives and information about the barriers and facilitators of postpartum contraceptive access and use within this population.

#### Concept

The development of the review questions was guided by the World Health Organization’s definition of postpartum family planning, as “services for the prevention of unintended pregnancy and closely spaced pregnancies through the first 12 months following childbirth” [2]. This definition is similar to that used by the ACOG: “using a birth control method in the weeks after [having a baby] to avoid an unintended pregnancy…experts recommend waiting at least 18 months between pregnancies before having another baby” [33]. Data will include postpartum family planning and contraceptive information and counseling, patterns of use, and contraceptive methods. There will be no restriction on the types of contraceptive methods reported in eligible studies. Thus, eligible sources will include studies on very effective methods such as sterilization, long acting reversible contraceptives (LARCs) such as IUDs and implants; effective methods such as short-acting hormonal contraceptives, including the pill, injectable, emergency contraception, and lactational amenorrhea method (LAM); moderately effective methods such as barrier methods, for example, the female and male condoms, and fertility awareness methods; and less effective methods, such as, withdrawal or coitus interruptus [34]. We will also identify key barriers, facilitators, and social determinants, of postpartum contraception access and utilization in sub-Saharan African immigrant and refugee women. Some of these include, access to insurance coverage, literacy levels, language, patient-provider factors, childcare, transportation, stigma, and knowledge/awareness of available/preferred methods [9,24,35].

#### Context

Only studies conducted in, and on participants currently residing in the United States will be included in this review. Studies of sub-Saharan African immigrant and refugee women residing outside the United States will be excluded. Only full-text peer-reviewed studies will be included in this review. Further, only papers written in English will be included. Conference abstracts will be excluded.

### Exclusion criteria

We will exclude studies that do not include sub-Saharan African immigrant and refugee women of reproductive age (i.e., 15–49 years). Sources that include immigrant and refugee populations born in countries outside of the sub-Saharan Africa region will also be excluded. Studies that are conducted with populations living outside the United States, studies that were published prior to 2000, and in a language other than English will also be excluded. Finally, sources that do not include postpartum contraception will also be excluded from this scoping review.

### Types of studies

All types of study designs will be considered in this review, including randomized controlled trials (RCTs), non-RCTs, pre/post intervention and time series designs. Observational studies, including prospective, retrospective, cross-sectional, descriptive, and case studies will also be included in the review. We will include systematic and other types of reviews. Grey literature such as technical reports and program evaluation reports will also be considered. We will include opinion papers in this scoping review. Across all the aforementioned types of sources, we will include quantitative, qualitative, and mixed methods designs in this review.

### Theoretical framework

The PEN-3 Model, which centers culture in the understanding of health behaviors and outcomes, [36], will be used as a guiding framework for this study. The PEN-3 Model uses a strength-based approach to identify individual, family and community values and assets, as well as social and cultural norms, and factors that may facilitate or hinder behaviors that affect health outcomes [36,37]. Since its introduction in the context of studying the impact of the sociocultural context on health behaviors in sub-Saharan Africa, the model has been used extensively in other areas of research and programs, including SRH, adolescent health, family planning, and chronic disease prevention and management in several countries [36,38–40]. The model has three domains, each with three constructs, namely: 1) cultural identity (constructs: person, extended family, neighborhood); 2) relationships and expectations (constructs: perceptions, enablers, and nurturers; and 3) cultural empowerment (constructs: positive, unique/existential, and negative factors).

### Search strategy

A public health research librarian helped the authors with identifying keywords and developing the preliminary search terms, including Boolean search terms, and relevant databases for an exploratory database search in PubMed/MEDLINE (Table 1). We will search the following databases: PubMed/MEDLINE, PsycINFO, CINAHL, EMBASE, and the Global Health Database. In addition, we will search the grey literature, including the webpages of multilateral organizations such as the World Health Organization and United Nations agencies, as well as the Migration Policy Institute, the American College of Obstetricians and Gynecologists, and the Centers for Disease Control and Prevention. We will also conduct a manual search of article reference lists in Google Scholar to ensure we capture the breadth of the available research on the topic. Further, we will manually search the publications of key reproductive health journals for relevant sources that may not be retrieved in our database search. Prior to the implementation of the review, we will work with the professioanal librarian to update and refine the search terms as needed. All changes to the protocol will be highlighted in the reporting of the review, when completed.

**Table 1:** PubMed/Medline preliminary search strategy.

Descriptors	Population: sub-Saharan African immigrants and refugees	AND	Concept: Postpartum contraception	AND	Context: United States
	(immigrants or immigration or immigrant or refugee or migrant or refugees or refugee women) OR (emigrants and immigrants[mesh]) OR immigrant OR (sub saharan africa or sub-saharan africa or sub sahara or sub-sahara or ssa) OR (West Africa or East Africa* or South Africa* or Central Africa or Middle Africa*)		("birth intervals/ethnology"[MeSH Terms] OR "birth intervals/psychology"[MeSH Terms] OR "birth intervals/statistics and numerical data"[MeSH Terms]) AND "Contraception"[MeSH Terms] "birth intervals"[MeSH Terms] AND "Contraception"[MeSH Terms] "birth intervals"[MeSH Terms] AND "Contraception"[MeSH Terms] (contraception or birth control or family planning or contraceptive or pregnancy prevention) OR (postpartum period OR (postnatal or postpartum) OR (interpregnancy interval or pregnancy interval or birth spacing or inter-birth interval or interbirth interval)		United States OR U.S. or US or USA or America*

### Study screening and selection process

The search will cover the period from January 1, 2000, to June 30, 2023. We selected this period for the search because sub-Saharan African immigration to the US has increased rapidly since 2000 [8]. When the database searches are completed, we will review the reference lists of selected articles to identify additional, relevant sources. The final list of relevant sources will be imported into Covidence, which will automatically identify duplicates for removal. Covidence will be used to manage all steps of the title/abstract and full-text screening. We will review the titles and abstracts of the retrieved results and exclude those that do not meet the inclusion criteria. Abstract and title screening will be carried out independently by two reviewers. Where there are disagreements between both reviewers, these will be resolved by discussion. In a second screening step, full texts of the retained articles will be imported into Covidence, via Zotero, a bibliography management software, [41]. Detailed assessment of the full texts of articles will be independently carried out by two reviewers. Full texts not meeting inclusion criteria will be deleted in this step. Where there are disagreements among the two reviewers, these will be resolved by consensus or a third reviewer if warranted. The full-text articles to be included in the data synthesis and analysis will be finalized. We will develop a PRISMA flowchart to document the bases for inclusion or exclusion of articles [42].

### Data extraction and charting

Using the JBI (Joanna Briggs Institute) data extraction manual as a guide [30], we developed a draft data extraction tool (Table 2). The data extraction tool will be modified and revised as needed during the data extraction process, to to capture pertinent information from each included source of evidence To pilot the data extraction tool, two reviewers will independently screen a subset of the full-text sources, using the draft data extraction tool to record key information from the subset of studies. We will include 10% of the total number of sources meeting the review includsion criteria in this pilot [43]. Upon finalization of the data extraction tool, each of the two reviewers will be assigned a subset from the remaining articles to review. The data extracted will include details about the participants’ sociodemographic information (e.g., age, education, parity, country of birth, immigration status), concept (e.g., postpartum contraceptive method use), and context (e.g., state or region of residence in the US) (PCC). We will also extract data about study details specific to each included source, such as authors’ names, article title, year of publication, study aims/objectives, study design, study population (country of birth), sample size, and key findings related to the review questions. The two reviewers will discuss discrepancies and necessary amendments to the data extraction tool. If needed, a subject matter expert will serve as a third reviewer to help resolve discrepancies.

**Table 2:** Preliminary data extraction tool.

Author(s) and year of publication
Article title
Aims/objectives
Study design/ sample size/methods
Key findings
Population
Concept
Context
Findings related to key review question(s)
Predisposing factors
Barriers
Facilitators

### Summarizing, analyzing, synthesizing, and reporting the data

The extracted data will be tabulated, accompanied by a narrative summary of the characteristics of the included studies, for example, publication date, study participants’ country of origin, and study design. Table 2 outlines the key information that will be extracted. We will present the results quantitatively using frequencies, and qualitatively using thematic analysis. The results will be further analyzed using narrative synthesis, which is a suitable approach for systematically synthesizing evidence from multiple sources using words and text to summarize and explain the findings [44]. We will identify social and structural factors that may influence postpartum contraception and family planning. These findings will be organized into perceptions, enabling, and nurturing factors, and will then, be categorized into positive, unique/existential, and/or negative factors, using the PEN-3 Model domain and constructs [37,38]. The data will be presented in tables and charts, accompanied by narrative descriptions of each table and chart.

### Quality assessment and risk of bias

Consistent with the JBI Manual for conducting scoping reviews [30], the risk of bias or methodologic quality of included studies will not assessed. To minimize the risk of bias, we have developed a plan for at least two reviewers to independently conduct the title/abstract and full text screening of retrieved sources. The exclusion of articles published in a language other than English is not expected to be a source of bias since the focus of this review is on the US, where the official language is English. The exclusion of studies published prior to 2000 may eliminate some sources; however, we expect this to cause minimal bias since sub-Saharan African immigration to the US only started to rise sharply in the 2000’s.

## Discussion

This paper presents a protocol for a scoping review aimed at mapping the existing research on postpartum contraception among sub-Saharan African immigrant and refugee women living in the US. It has been observed that foreign-born Black women exhibit a less favorable attitude towards contraceptive uptake compared to their US-born counterparts [45]; hence it would be meaningful to assess if this correlation extends to contraceptive method use during the postpartum period. Additionally, contraceptive care for resettled refugees is reported to be limited due to the absence of epidemiological data on their distinctive needs [35].

The primary objective of this review is to identify knowledge gaps and utilize existing evidence to support policies, programs, and advocacy for reducing the prevalence of short-interval births among sub-Saharan African immigrants. Findings will be discussed in the context of previous research. We will also discuss the results of this scoping review in relation to future research, policy, and service delivery to support African-born migrant and refugee women living in the US, to prevent unintended pregnancy and achieve healthy birth intervals and reproductive autonomy.

Short interpregnancy interval is associated with adverse maternal outcomes, such as increased risks of subsequent obesity, gestational diabetes, precipitous labor, and placental abruption, as well as decreased risks of preeclampsia and labor dystocia. Furthermore, a previous caesarean delivery followed by a short interval pregnancy of less than 6 months has been linked to increased risks of uterine rupture, blood transfusions, and other birthing complications [46].

## Limitations and strengths

As a scoping review’s focus is to provide a broad overview of the subject matter, a limitation is that an in-depth analysis may be hampered. We also acknowledge the challenge in ensuring that the search strategy is neither too narrow nor too broad. The wide range of study designs and methodologies that will be included may lead to heterogeneity among the studies, making it challenging to synthesize the findings. This limitation will be mitigated by using a narrative synthesis approach for analyzing the retrieved data [44]. Additionally, the methodological quality of the included studies will not be assessed. It should be noted that, consistent with guidelines [30], quality assessment of retrieved studies is not a focus of scoping reviews.

Despite these limitations, scoping reviews play a vital role in research by offering an overview of existing literature, identifying knowledge gaps that may warrant a systematic review, and outlining areas for future research. The findings of this scoping review will be disseminated through a peer-reviewed journal and presented at national/international conferences. This scoping review will contribute to the existing knowledge on, and aid in the development of evidence-based interventions for promoting postpartum contraception among African immigrant and refugee women living in the US.

### Potential contributions of this scoping review

Through this review and data synthesis, we aim to propose a working understanding of the concept of postpartum contraception among sub-Saharan African immigrant and refugee women. The review will provide insights into various aspects, including postpartum contraceptive information and counseling, patterns of use, and the range of contraceptive methods utilized. Additionally, we will identify barriers and facilitators related to postpartum contraceptive education and counseling, and contraceptive access (e.g., insurance, childcare, transportation,), as well as health system and structural barriers/facilitators (e.g., preferred/available methods, language, provider factors, etc.). This review, when completed, will be published in a peer-reviewed journal e.g., *PLoS ONE*. Findings will also be disseminated to knowledge users via scientific conference presentations and seminars.

## Supporting information

S1 File PRISMA 2020 checklist. (DOCX)

## Abbreviations

ACOG: American College of Obstetricians and Gynecologists
IPI: Interpregnancy interval
JBI: Joanna Briggs Institute
LAM: Lactational amenorrhea method
LARC: Long-acting reversible contraceptive
PCC: Population, Concept, Context
PRISMA-ScR: Preferred Reporting Items for Systematic Reviews and Meta-Analyses, extension for scoping review
SRH: Sexual and Reproductive Health
WHO: World Health Organization
