# Supplementary material for: Mapping the evidence on factors related to postpartum contraception among sub-Saharan African immigrant and refugee women in the United States of America: A scoping review protocol
Source: PLoS One. 2024 May 29;19(5):e0304222. doi: 10.1371/journal.pone.0304222 (PMC11135752; doi:10.1371/journal.pone.0304222)
Supplement: S1 File — (DOCX) [file pone.0304222.s001.docx]

**Preferred Reporting Items for Systematic reviews and Meta-Analyses extension for Scoping Reviews (PRISMA-ScR) Checklist**

| SECTION | ITEM | PRISMA-ScR CHECKLIST ITEM | REPORTED ON PAGE # |
| --- | --- | --- | --- |
| TITLE | | | |
| Title | 1 | Mapping the evidence on factors related to postpartum contraception among sub-Saharan African immigrant and refugee women in the US: A scoping review protocol | 1 |
| ABSTRACT | | | |
| Structured summary | 2 | **Background**  Postpartum contraception is essential to sexual and reproductive health (SRH) care because it encourages healthy spacing between births, helps women avoid unwanted pregnancies, and lessens the risks of health problems for mothers and babies. Sub-Saharan African immigrant and refugee populations are rapidly increasing in the United States, and they come from a wide range of cultural, linguistic, religious, and social origins, which may pose challenges in timely access to culturally acceptable SRH care, for preventing mistimed or unwanted childbearing. The objective of this scoping review is to assess the extent of the available literature on postpartum contraception among sub-Saharan African immigrant and refugee women living in the United States.  **Methods**  We developed preliminary search terms with the help of an expert librarian, consisting of keywords including birth intervals, birth spacing, contraception, postpartum contraception or family planning, and USA or America, and sub-Saharan African immigrants, or emigrants. The study will include the following electronic databases: PubMed/MEDLINE, PsycINFO, CINAHL, EMBASE, and the Global Health Database. The sources will include studies on postpartum care and contraceptive access and utilization among sub-Saharan African immigrants living in the US. Citations, abstracts, and full texts will be independently screened by two reviewers. We will use narrative synthesis to analyze the data using quantitative and qualitative methods. Factors associated with postpartum contraception will be organized using the domains and constructs of the PEN-3 Model as a guiding framework.  **Discussion Conclusion**  This scoping review will map the research on postpartum contraception among sub-Saharan African immigrant and refugee women living in the US. We expect to identify knowledge gaps, and barriers and facilitators of postpartum contraception among immigrant and refugee women in this population. Based on the findings of the review, recommendations will be made for advocacy and program and policy development toward optimizing interpregnancy intervals in sub-Saharan African immigrants living in the US. | 2 |
| INTRODUCTION | | | |
| Rationale | 3 | Postpartum contraception is essential to sexual and reproductive health (SRH) care because it helps women avoid unwanted pregnancies, lessens the risk of health problems for mothers and babies, and encourages health spacing between births (1). Sub-Saharan African immigrant and refugee populations are rapidly increasing in the United States, and they come from a wide range of cultural, linguistic, religious, and social origins (7). They may have trouble getting postpartum contraception and other preventative SRH care because of their prior experiences with SRH in their home countries, relocation, migration, and resettlement experiences (8). Evidence from other comparable migrant-receiving nations (European Union and Asia) (9) and a recent systematic review study of five countries (10) have revealed many factors that contribute to low rates of maternal health treatment usage among immigrant women. Understanding the factors influencing postpartum contraceptive use among sub-Saharan African immigrant and refugee women is vital for developing targeted interventions that address their unique needs. Sub-Saharan African immigrant and refugee women face various challenges; culture and personal beliefs largely influence decisions regarding postpartum contraception. Some cultural norms and religious beliefs discourage modern contraceptive methods, endorsing more traditional methods or no contraception at all. Access to healthcare services is a significant structural factor affecting postpartum contraception use among these women. These barriers can limit these women's ability to receive timely contraceptive counseling and access contraceptive services. To our knowledge, there is no comprehensive review of the evidence on factors related to postpartum contraception among sub-Saharan African immigrant and refugee women living in the US. | 3-6 |
| Objectives | 4 | The purpose of this scoping review is to map the evidence on factors related to postpartum contraception among sub-Saharan African immigrant and refugee women residing in the US. The review will identify barriers and facilitators to postpartum family planning services among immigrant and refugee people living in the US. By identifying the key factors influencing postpartum contraceptive use in this population, this study, when completed, can inform the development of interventions and policies promoting reproductive health equity for immigrant and refugee women.  The questions we seek to answer via this scoping review are:  Primary Review Question: What is the evidence on postpartum contraception among sub-Saharan African immigrant and refugee women living in the US? In addition, three sub questions that will enable the research team further explore specific attributes of the review’s population, concept, and context (PCC) will be explored, as outlined below:   - What immigrant populations are included in the research reported in the literature? - What are the barriers to and facilitators of postpartum contraception from the perspectives of sub-Saharan African immigrant and refugee women and health care providers in the US? - What is the evidence linking postpartum contraceptive counseling and use to the prevention of short-interval births or unintended pregnancy? | 6 |
| METHODS | | | |
| Protocol and registration | 5 | A review protocol has been developed and submitted to Open Science Framework for registration. The protocol is accessible at: https://osf.io/s385j. | 7 |
| Eligibility criteria | 6 | **Participants:** Eligible participants will include sub-Saharan African immigrant and refugee women living in the US. Studies will include those on women who have had at least one live birth. Other criteria include studies that used data on health care providers’ (nurses, family physicians, obstetrician/gynecologists, midwives, doulas, social workers, refugee resettlement workers, and other relevant key informants’ perceptions and information on barriers and facilitators of PP contraceptive use.  **Concept:** The main is postpartum contraception among sub-Saharan African immigrant and refugee women. This includes data on postpartum contraceptive information and counseling, patterns of use, methods (any), including lactational amenorrhea methods (LAM). Additional concepts include barriers and facilitators of postpartum contraceptive education and counseling, contraceptive access (e.g., insurance), health system and social barriers/facilitators (preferred/available methods, language, provider factors, childcare, transportation, stigma, etc.)  **Context:** Studies that describe postpartum contraception patterns, barriers and facilitators among sub-Saharan African immigrant and refugee women will be included in the review, as well as studies conducted in, and on participants living in the US. Studies on sub-Saharan African immigrants and refugees living in other countries will be excluded as will be studies conducted outside of the US or solely on immigrant and refugee groups other than those from sub-Saharan Africa, and conference abstracts.  Studies published between January 1, 2000 and June 30, 2023 will be included. We selected this period because of the rapid growth in sub-Saharan African immigration to the US since 2000. Eligible studies will be limited to those published in English. All types of study designs will be considered in this review, including quantitative, qualitative, and mixed methods designs. Peer-reviewed articles and grey literature, such as program technical reports, will be included in the review. | 7-8 |
| Information sources* | 7 | A public health research librarian will help the authors with identifying keywords and developing the preliminary search terms, including Boolean search terms, and relevant databases. We will search the following databases: PubMed/MEDLINE, PsycINFO, CINAHL, EMBASE, and the Global Health Database. In addition, we will search the gray literature, including the webpages of multilateral organizations such as the World Health Organization and United Nations agencies; as well as the Migration Policy Institute, the American College of Obstetricians and Gynecologists, and the Centers for Disease Control and Prevention.  We will also conduct a manual search of article reference lists in Google Scholar to ensure we capture the breadth of the available research on the topic. The search will be restricted to sources published in English. Prior to the implementation of the review, we will work with the subject librarian to refine and update the search terms as needed. | 10 |
| Search | 8 | **Trial search strategy for PubMed/MEDLINE (January 1, 2000 - June 30, 2023)**   \| **Descriptors** \| **Population: sub-Saharan** African immigrants and refugees \| **AND** \| **Concept:** Postpartum contraception \| **AND** \| **Context:** United States \| \| --- \| --- \| --- \| --- \| --- \| --- \| \| Keywords/ search terms \| ( immigrants or immigration or immigrant or refugee or migrant or refugees or refugee women ) OR ( emigrants and immigrants[mesh] ) OR immigrant OR ( sub saharan africa or sub-saharan africa or sub sahara or sub-sahara or ssa ) OR ( West Africa or East Africa* or South Africa* or Central or Middle Africa* ) \|  \| ("birth intervals/ethnology"[MeSH Terms] OR "birth intervals/psychology"[MeSH Terms] OR "birth intervals/statistics and numerical data"[MeSH Terms]) AND "Contraception"[MeSH Terms]  "birth intervals"[MeSH Terms] AND "Contraception"[MeSH Terms]  "birth intervals"[MeSH Terms] AND "Contraception"[MeSH Terms]  ( contraception or birth control or family planning or contraceptive or pregnancy prevention )  OR (postpartum period OR ( postnatal or postpartum ) OR ( interpregnancy interval or pregnancy interval or birth spacing or inter-birth interval or inter-birth interval ) \|  \| United States OR  U.S. or US or USA or America* \| | 12-13 |
| Selection of sources of evidence† | 9 | The retrieved results will be imported, via a Zotero bibliography management software, into Covidence, which will automatically remove duplicates. Two reviewers will independently conduct title and abstract screening. Eligible full texts will be screened independently by two reviewers. Where there are disagreements among the two reviewers, these will be resolved by consensus or a third reviewer if warranted. The full-text articles to be included in the review will be finalized. | 13 |
| Data charting process‡ | 10 | Guided by the JBI data extraction manual, a first reviewer will develop the data extraction form. The data extraction tool will be tailored to the needs of the proposed study to capture pertinent information from the articles to be included. This will include authors’ names, article title, year of publication, study aims/objectives, study design, study population (country of birth), sample size, and concept and key findings related to the review questions. We will share the form with a second reviewer to ensure it will adequately capture the required data. Both reviewers will independently screen a subsample of full texts and extract the data. The reviewers will discuss discrepancies and necessary amendments to the form. If needed, a third reviewer will be invited to help resolve discrepancies and finalize the data extraction tool. Both reviewers will, then, proceed to extract and chart the data from the remaining articles. | 13-14 |
| Data items | 11 | Sociodemographic and cultural factors related to postpartum contraception, including barriers and facilitators will be elucidated. Other data items will include type of study design, publication date, and authors. | 14 |
| Critical appraisal of individual sources of evidence | 12 | We will not conduct a critical appraisal of included sources of evidence as this is a scoping review. | 15 |
| Synthesis of results | 13 | We will use quantitative methods, including frequencies and percentages to summarize and present the results. Additionally, we will use thematic analysis to qualitatively summarize the results. Further, the results will be further analyzed using narrative synthesis, which is a suitable approach for systematically synthesizing evidence from multiple sources using words and text to summarize and explain the findings. | 14 |
| RESULTS | | | |
| Selection of sources of evidence | 14 | Not applicable | Click here to enter text. |
| Characteristics of sources of evidence | 15 | Not applicable | Click here to enter text. |
| Critical appraisal within sources of evidence | 16 | Not applicable | Click here to enter text. |
| Results of individual sources of evidence | 17 | Not applicable | Click here to enter text. |
| Synthesis of results | 18 | Not applicable | Click here to enter text. |
| DISCUSSION | | | |
| Summary of evidence | 19 | This scoping review will contribute to the existing knowledge on postpartum contraception among sub-Saharan African immigrant and refugee women living in the US. The results will be disseminated through a peer-reviewed manuscript and may be presented at scientific conferences and via seminars. | 14-16 |
| Limitations | 20 | One of the limitations of this scoping review is ensuring that the search strategy is neither too narrow nor too broad. The wide range of study designs and methodologies that will be included may lead to heterogeneity among the sources. As noted earlier, the methodological quality of the included studies will not be assessed as quality assessment is not a focus of scoping reviews. | 17 |
| Conclusions | 21 | The primary objective of this review is to analyze the existing research and identify knowledge gaps on postpartum contraception among African immigrant and refugee populations living in the US. The results may contribute to supporting policies, programs, and advocacy for reducing the prevalence of short-interval births in this population. | 17-18 |
| FUNDING | | | |
| Funding | 22 | There is no funding associated with this study. | Click here to enter text. |

JBI = Joanna Briggs Institute.

* Where sources of evidence (see second footnote) are compiled from, such as bibliographic databases, social media platforms, and Web sites.

† A more inclusive/heterogeneous term used to account for the different types of evidence or data sources (e.g., quantitative and/or qualitative research, expert opinion, and policy documents) that may be eligible in a scoping review as opposed to only studies. This is not to be confused with information sources (see first footnote).

*From:* Tricco AC, Lillie E, Zarin W, O'Brien KK, Colquhoun H, Levac D, et al. PRISMA Extension for Scoping Reviews (PRISMAScR): Checklist and Explanation. Ann Intern Med. 2018;169:467–473. [doi: 10.7326/M18-0850](http://annals.org/aim/fullarticle/2700389/prisma-extension-scoping-reviews-prisma-scr-checklist-explanation).
